# The Diverse Pathways for Cell Surface MT1-MMP Localization in Migratory Cells

**DOI:** 10.3390/cells14030209

**Published:** 2025-01-31

**Authors:** Hannah Kelly, Masaki Inada, Yoshifumi Itoh

**Affiliations:** 1 The Kennedy Institute of Rheumatology, Nuffield Department of Orthopaedics, Rheumatology and Musculoskeletal Sciences, University of Oxford, Oxford OX3 7FY, UK; hannah.kelly@hertford.ox.ac.uk; 2 Department of Biotechnology and Life Science, Tokyo University of Agriculture and Technology, 2-24-16 Nakacho, Koganei, Tokyo 184-8588, Japan; inada@cc.tuat.ac.jp; 3 Institute of Global Innovation Research, Tokyo University of Agriculture and Technology, 2-24-16 Nakacho, Koganei, Tokyo 184-8588, Japan

**Keywords:** MT1-MMP, cell invasion, leading-edge, focal adhesion, invadopodia

## Abstract

Controlled cell migration is an essential biological process in health, while uncontrolled cell migration contributes to disease progression. For cells to migrate through tissue, they must first degrade the extracellular matrix (ECM), which acts as a physical barrier to cell migration. A type I transmembrane-type matrix metalloproteinase, MT1-MMP, is the key enzyme involved in this process. It has been extensively shown that MT1-MMP promotes the migration of different cell types in tissue, including fibroblasts, epithelial cells, endothelial cells, macrophages, mesenchymal stem cells, and cancer cells. MT1-MMP is tightly regulated at different levels, and its localization to leading-edge membrane structures is an essential process for MT1-MMP to promote cellular invasion. Different cells display different motility-associated membrane structures, which contribute to their invasive ability, and there are diverse mechanisms of MT1-MMP localization to these structures. In this article, we will discuss the current understanding of MT1-MMP regulation, in particular, localization mechanisms to these different motility-associated membrane structures.

## 1. Introduction

Cell migration is a fundamental process, which plays a role in various pathophysiological conditions. In physiological conditions, cell migration is tightly regulated and plays crucial roles in development, wound healing, and immune responses. In pathological situations, such as cancer and rheumatoid arthritis (RA), cell migration becomes uncontrolled and contributes to disease progression. This uncontrolled cell migration is often associated with excessive tissue destruction, leading to organ malfunction. Tissues are made up of cells and extracellular matrix (ECM). Two different types of cell migration in tissues have been described: amoeboid migration and mesenchymal migration [1]. Amoeboid migration is a type of cell migration that alters cell shape to fit through pores in the ECM, which does not require ECM degradation. This requires ECM pores to be, at a minimum, the size of the cell nucleus, around 7 μm for most migratory cells [2]. If the ECM is too dense for cells to migrate using amoeboid migration, cells adopt a mesenchymal migration, which uses proteolytic degradation of the ECM to enlarge pore size and create a path for migration [3,4].

**Figure 1:**
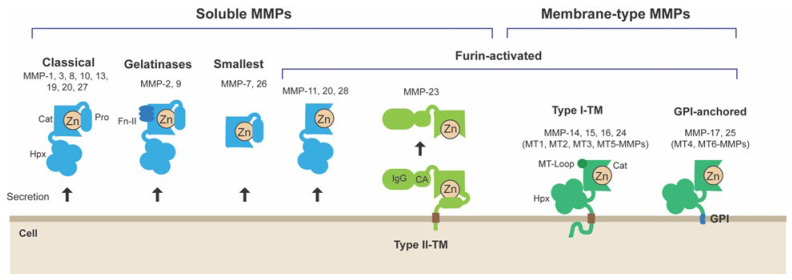
Matrix metalloproteinases (MMPs) can be divided into two subgroups of soluble and membrane-type MMPs. Soluble MMPs can be further classified into Classical, Gelatinases, Smallest, Furin-activated, and Type II-TM MMPs. The typical domain structure contains a prodomain (Pro), a catalytic domain (Cat), a hinge, and a hemopexin domain (Hpx). Gelatinases also contain three repeats of a fibronectin-type II domain insert (FN-II). The smallest MMPs lack the hemopexin domain. Furin-activated MMPs are activated during secretion by proprotein convertases such as furin. Type II-TM MMP-23 is classified as a soluble MMP, as it becomes a soluble enzyme upon intracellular activation by proprotein convertases. Membrane-type MMPs (MT-MMPs) can be classified into Type I-TM and GPI-anchored MT-MMPs. MT-MMPs are activated intracellularly by proprotein convertases. Type I -TM MT-MMPs have an insertion of MT-Loop in the catalytic domain, transmembrane domain, and a short cytosolic tail. GPI-anchored MT-MMPs are anchored to the plasma membrane through a GPI-anchor.

A major group of enzymes involved in ECM degradation is the matrix metalloproteinases (MMP), a family of zinc-dependent metalloendopeptidases [5]. MMPs are a family of 23 enzymes in humans; they can be divided into soluble- and membrane-type MMPs (Figure 1) [6]. Soluble MMPs are secreted to the extracellular milieu, while membrane-type MMPs are physically anchored to the plasma membrane (Figure 1). The location of the enzymes significantly impacts their biological functions. For instance, soluble-type MMPs can diffuse to even the acellular parts of the tissue, while the activities of membrane-type MMPs are restricted to the cell surface. MMPs, in general, have been considered to play roles in ECM degradation in different pathophysiological conditions, although in vivo functions of each enzyme have not been understood completely. However, it is becoming clear that MT1-MMP is the enzyme responsible for cellular ECM degradation found in different pathological processes [7,8]. MT1-MMP is a type I transmembrane proteinase expressed on the cell surface, and it degrades ECM in the direction of cell migration to promote cellular invasion [9]. Thus, its localization to leading-edge membrane structures is crucial. In this review, the roles and regulations of MT1-MMP are reviewed, and current knowledge on the localization of MT1-MMP to different leading-edge membrane structures is discussed.

## 2. MT1-MMP

**Figure 2:**
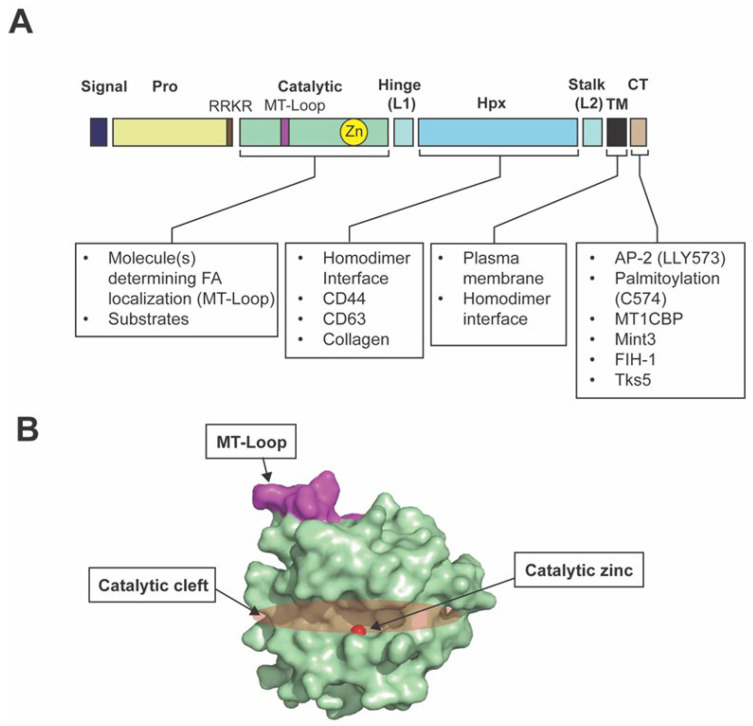
Domain structure of MT1-MMP and molecules that have been shown to interact with each domain structure. (**A**) The domain structure of MT1-MMP is indicated. The molecules shown to interact with each domain are also listed. (**B**) Surface representation of the MT1-MMP catalytic domain. The MT-Loop region is highlighted in purple. The catalytic zinc is shown in red. The catalytic cleft is highlighted in orange.

The domain structure of MT1-MMP consists of a signal peptide, a propeptide, a catalytic domain, a hinge or Linker-1, a hemopexin (Hpx) domain, a stalk or Linker-2, a transmembrane (TM) domain, and a short cytoplasmic tail (CT) of approximately 20 amino acids (Figure 2) [6]. AS MT1-MMP is a transmembrane protein, different domains of the MT1-MMP molecule have been shown to be necessary for its interactions with other molecules (Figure 2A). At the C-terminus of the propeptide, a basic motif Arg-Arg-Lys-Arg (RRKR) is present, which is recognized and cleaved by proprotein convertases (PCs) such as furin during secretion. MT1-MMP is, therefore, expressed on the cell surface in an active form. In the catalytic domain, an eight amino acid insertion of a ^163^PYAYIREG^170^ sequence, referred to as MT-loop, is present. This loop insert is only present in TM-type MT-MMPs and is located at the opposite position of the catalytic site within the catalytic domain (Figure 2B). It has been shown that the MT-Loop is essential for efficient proMMP-2 activation [10] and MT1-MMP localization to focal adhesions [11]. The Hpx domain has been shown to interact with CD44 to localize MT1-MMP to lamellipodia [12]. This domain was also shown to interact with the N-terminus of tetraspanin CD63 to target MT1-MMP to the lysosome upon endocytosis [13]. MT1-MMP forms a homodimer on the cell surface; the Hpx and transmembrane domains act as homodimerization interfaces [6]. The cytosolic tail of MT1-MMP is crucial for its clathrin-dependent endocytosis. The LLY^573^ motif within the cytosolic tail has been shown to bind the µ2 subunit of adaptor protein 2 (AP-2), which mediates the incorporation of MT1-MMP into clathrin cages [6]. Palmitoylation of the cytosolic tail at Cys^574^ was also found to be essential for clathrin-mediated endocytosis of MT1-MMP [6]. MT1-MMP cytoplasmic tail binding protein-1 (MTCBP-1) was identified as a binding protein to the cytosolic tail of MT1-MMP by yeast two-hybrid screening [14]. It is a member of the Cupin superfamily proteins and has been shown to negatively regulate MT1-MMP function [14,15,16].

MT1-MMP is expressed in different cell types, including fibroblasts, chondrocytes, osteoclasts, neurons, macrophages, lymphocytes, epithelial cells, endothelial cells, and cancer cells [6]. It is involved in various pathophysiological processes, such as wound healing, skeletal development, physiological and pathological angiogenesis, cancer cell invasion and metastasis, tumor growth, and cartilage erosion in rheumatoid arthritis [6]. MT1-MMP-null mice showed severe phenotypes of craniofacial abnormalities, osteoclast-mediated arthritis, osteopenia, soft tissue fibrosis, and dwarfism [17,18]. No other MMP-null mice showed such severe phenotypes.

### 2.1. MT1-MMP Functions

MT1-MMP is able to degrade a wide variety of ECM components, including fibrillar collagens type I, II, and III; fibronectin; vitronectin; laminins 1, 2, 4, and 5; and aggrecan core protein [6]. MT1-MMP is one of the five collagenase MMPs (MMP1, MMP2, MMP8, MMP13 and MT1-MMP) that can cleave fibrillar collagens, and is the only membrane-bound collagenase. It has been shown that MT1-MMP is the only enzyme capable of promoting cellular invasion in a collagen-rich matrix [19]. The membrane-bound nature of MT1-MMP is essential in promoting cellular invasion, and soluble MT1-MMP mutants could not promote invasion of Madin–Darby Canine Kidney (MDCK) epithelial cells [19].

MT1-MMP cannot directly degrade type IV collagen, a major component of the basement membrane. However, MT1-MMP can initiate the degradation of type IV collagen by activating proMMP2 on the cell surface [20]. Thus, the proMMP2 activation step is considered to be a crucial step in the initial invasion and growth of epithelial cancer [21]. The currently accepted model of proMMP2 activation is as follows. First, MT1-MMP forms a homodimer through the Hpx and transmembrane domains. Next, TIMP2 binds to one of the MT1-MMP catalytic domains in the dimer and recruits proMMP2 to the complex through the interaction between the proMMP2 Hpx domain and the TIMP2 C-terminal domain. Then, the free MT1-MMP molecule in the dimer that is not bound to TIMP2 cleaves the prodomain of MMP2 at Asn37-Leu [22]. Finally, the activated MMP2 is released [6,22,23]. MT1-MMP also initiates proMMP9 activation indirectly by activating MMP2, which then activates proMMP9 [24]. MT1-MMP can also activate proMMP13 on the cell surface [25]. Although the mechanism underlying this activation is not fully understood, it is known that the Hpx domain of proMMP13 is essential for this activation, and TIMP2 is not involved [26].

MT1-MMP cleaves different membrane proteins on the cell surface, including CD44, syndecan-1, ICAM1, and LRP-1 [27,28,29,30]. CD44 is a hyaluronan receptor, and its shedding by MT1-MMP was shown to promote cell migration and invasion [27]. In addition, MT1-MMP interaction with CD44 through the Hpx domain is required for the shedding of MT1-MMP from the cell surface [31]. Syndecan-1 is a transmembrane heparan sulphate proteoglycan. Shedding of its ectodomain at Ala243-Ser by MT1-MMP promotes cell migration [28]. MT1-MMP also sheds the cell adhesion molecule ICAM1 at the leading edge [29]. Shedding of LRP-1 by MT1-MMP has been shown to play a number of roles within different cell types; for example, LRP-1 shedding in chondrocytes increases cartilage degradation in osteoarthritis by preventing endocytosis of MMP13 and ADAMTS5 aggrecanase [32]. In cancer cells, LRP-1 shedding controls the fate of MMP2 and other soluble proteases [30], and in vascular smooth muscle cells, it helps to regulate dedifferentiation, which occurs in response to blood vessel wall injury [33].

MT1-MMP has also been shown to exert non-proteolytic functions. In macrophages and cancer cells, MT1-MMP was shown to cause the Warburg effect [34], that is, generating ATP through the glycolytic pathway even under normoxic conditions [35]. The cytoplasmic tail of MT1-MMP binds to FIH-1 (factor inhibiting HIF-1) (Figure 2A), allowing FIH-1 to be inhibited by Mint3/APBA3 [34]. This allows HIF-1α pathways be activated under normoxic conditions, causing hypoxic gene activation and enhanced glycolysis [36]. It has been shown that the expression of MT1-MMP can induce the Warburg effect, whereas the knockdown of MT1-MMP decreases lactate production by 50% in MDA-MB-231 cells [36]. Therefore, MT1-MMP is an important modulator of tumor cell metabolism. This aspect has been extensively discussed in a recent review [37].

Other non-proteolytic functions of MT1-MMP include stimulating VEGF-A production to promote angiogenesis; activation of the phosphoinositide-3-kinase-d (PI3Kd)/Akt/GSK3b signalling cascade in macrophages, which regulates macrophage immune response; regulating Notch signalling in bone marrow stromal cells; and facilitating myoblast fusion through fibronectin degradation [38,39,40,41]. For a more in-depth discussion of these functions, please see Reference [6].

### 2.2. Cellular Regulation of MT1-MMP

#### 2.2.1. Gene Expression

The promotor region of MT1-MMP includes the Sp1, Egr-1, AP-4, and Nkx-2 binding sites within -2kb of its translation start site [42,43]. The Sp1 binding site overlaps with the Egr-1 binding site and plays a role in collagen-induced MT1-MMP gene expression [43]. Additionally, the transcription factor Snail1, a regulator of epithelial–mesenchymal transition (EMT), has been associated with MT1-MMP-induced cell invasion of carcinoma cells [44]. Transformation of normal MDCK cells with *v-src* causes EMT, accompanied by the overexpression of MT1-MMP and enhanced cellular invasiveness [45]. These findings suggest that EMT is one of the major stimuli for MT1-MMP expression in cancer. On the other hand, MT1-MMP gene expression is not well-regulated by cytokines and growth factors as, unlike many soluble MMPs, its promotor region lacks a TATA box, AP1, AP2, and TGFβ binding sites [42,43]. The MT1-MMP promotor region does contain NFkB binding sites, but these are distant from the translation start sites, so NFkB-activating stimuli, such as TNFα, do not have a significant effect on MT1-MMP gene expression [43].

One of the potential in vivo stimuli of MT1-MMP expression is fibrillar collagen. It has been shown that type I collagen induces the expression of MT1-MMP in different cell types, such as fibroblasts, endothelial cells, epithelial cells, and some cancer cells [46,47,48,49]. In fibroblasts, it has been shown that collagen types I and II stimulate MT1-MMP gene expression and function by activation of a collagen receptor tyrosine kinase, discoidin domain receptor 2 (DDR2) [50]. However, collagen-mediated MT1-MMP expression in HT1080 fibrosarcoma cells was shown to be DDR2-independent, suggesting that the involvement of DDR2 is specific to fibroblasts [50]. In cancer cells, integrins, such as αvβ3, have been shown to mediate collagen-induced MT1-MMP expression [49].

A transcriptional factor, Krüppel-like factor 6 (KLF6), has been shown to be important for injury-induced MT1-MMP expression in endothelial cells [51], whereas serum amyloid A activating factor-1 (SAF1) has been shown to upregulate MT1-MMP in macrophages present in atherosclerotic plaques [52]. HIF-2α and SP1 promote MT1-MMP expression in Von Hippel–Lindau renal cell carcinoma (VHL RCC) [42], while in gastric cancer, myeloid zinc finger 1 (MZF1) has been shown to upregulate MT1-MMP expression [53].

#### 2.2.2. Homodimer Formation

MT1-MMP forms a homodimer through both the Hpx and the transmembrane domains [6]. Hpx domain-mediated dimerization is essential for cell surface collagen degradation, while transmembrane domain-mediated dimerization is required for proMMP-2 activation [54]. Expression of a dominant-negative mutant enzyme (a catalytic domain-deletion mutant of MT1-MMP), which inhibits dimerization of full-length MT1-MMP, effectively inhibited proMMP-2 activation and cell surface collagen degradation [55,56]. In HT1080 fibrosarcoma cells migrating in a 3D collagen matrix, the MT1-MMP dimer was consistently found at the leading edge and was shown to be regulated by the reorganization of fibrillar actin by Rac1 small GTPase [57]. Therefore, dimerization is considered to be a functional activation step of MT1-MMP, which is regulated according to fibrillar actin reorganization during cellular invasion.

#### 2.2.3. Leading-Edge Localization 

Another important regulatory step in MT1-MMP expression is its localization at the leading edge of migrating cells. MT1-MMP localizes to lamellipodia [55,58], focal adhesions [59,60], invadopodia [61,62], and podosomes [61,62]. These membrane structures are considered to be either the leading edge or the precursor of the leading edge in migratory cells. When cells migrate, ECM degradation should occur only at the leading edge, since the ECM is an important scaffolding for cell migration. Since MT1-MMP dimerization always occurs at the leading edge, as discussed above, the localization of MT1-MMP at these membrane structures can be considered as another step of its functional activation.

#### 2.2.4. Endocytosis

After cell surface expression, MT1-MMP is cleared from the cell surface by endocytosis, and some of the endocytosed MT1-MMP molecules are recycled back to the cell surface [63]. It has been shown that MT1-MMP can be endocytosed by at least three different pathways, specifically clathrin-mediated [64], caveolin-mediated [63], and flotillin-mediated pathways [65]. Clathrin-mediated endocytosis is a quicker process than caveolin-mediated endocytosis (5–30 min compared to >60 min) [63,66]. In clathrin-mediated endocytosis, the cytoplasmic domain of MT1-MMP is essential as the LLY^573^ motif in the cytoplasmic tail of MT1-MMP interacts with µ2 subunit of the adaptor protein 2 (AP2) complex, a component of clathrin cages [64]. Inhibition of the clathrin pathway by mutating LLY^573^ to AAA has been shown to abrogate MT1-MMP-mediated enhanced cell migration [64]. Thus, the endocytic process is essential for MT1-MMP to promote cell migration and invasion. MT1-MMP is palmitoylated at Cys^574^, located just downstream of the LLY^573^ motif in the cytosolic tail. Inhibition of the palmitoylation by mutating Cys^574^ to Ala abrogated clathrin-mediated endocytosis of MT1-MMP, and MT1-MMP-mediated promotion of cell migration [66]. It was also shown that Thr^567^ phosphorylation by protein kinase C is required for clathrin-mediated MT1-MMP endocytosis [67].

Caveolae-mediated endocytosis is another major pathway of MT1-MMP endocytosis [63,64,68,69]. It has been shown that this is the major endocytic pathway for MT1-MMP in endothelial cells [68]. Reduction in caveolin-1 expression in endothelial cells was shown to decrease MT1-MMP internalization and cell surface gelatin-degrading activity. However, the caveolae-mediated endocytosis pathway seemed to be less active when cells were cultured on a type I collagen matrix [68].

Flotillins (1 and 2) are known to mediate another clathrin-independent endocytic process. Flotillins have a similar molecular topology to caveolin 1 and generate membrane structures resembling caveolae [70]. Flotillin-mediated endocytosis of MT1-MMP has been demonstrated in breast carcinoma MDA-MB-231 cells and Rh41 rhabdomyosarcoma cells [65]. In both of these cell lines, Flotillin 1 and 2 were highly expressed, and the knockdown of flotillins significantly decreased collagen invasion. In normal non-invasive MCF10A mammary epithelial cells and C2C12 osteoblasts cells, expression of flotillins was low, and ectopic expression of flotillin 1 and 2 significantly increased collagen invasion of these cells. The flotillin-mediated endocytic vesicles are transported to the endolysosome, which is required for the exocytosis of MT1-MMP to the invadopodia [65].

Upon endocytosis, some MT1-MMP molecules are targeted for lysosomal degradation. It has been shown that the interaction of MT1-MMP with tetraspanin CD63, through the Hpx domain of the enzyme and N-terminal region of CD63, is crucial in this process [13]. MT1-MMP can also be recycled back to the cell surface after endocytosis [63].

## 3. Mechanisms of MT1-MMP Localization to the Leading-Edge Membrane Structures

As discussed above, one of the main cellular regulatory mechanisms of MT1-MMP is its localization to the leading-edge membrane structures of migrating cells. It has been shown that MT1-MMP localizes to different motility-associated membrane structures in migrating cells, including lamellipodia, focal adhesion, invadopodia, and podosomes, to facilitate cell invasion. However, it appears that different cells use different membrane structures for ECM degradation and invasion. Table 1 lists the cell types reported to use focal adhesion, invadopodia, and podosomes for ECM degradation. In this section, the current knowledge of the localization of MT1-MMP to each membrane structure is discussed.

### 3.1. Localization at Lamellipodia

In motile cells, MT1-MMP is highly localized at lamellipodia/ruffling membrane structures [55,58]. One of the mechanisms of MT1-MMP localization at the lamellipodia/ruffling membrane is its binding to CD44 through the Hpx domain of the enzyme [12]. CD44 is known to localize at lamellipodia and bind ERM proteins at its cytoplasmic domain, which connects it to F-actin [81]. Forming a complex with CD44, therefore, indirectly associates MT1-MMP with the actin cytoskeleton [12]. MT1-MMP was also found to interact with ERM proteins at its cytoplasmic domain [82,83], allowing MT1-MMP to associate with F-actin through ERM proteins without associating with CD44. However, it is not understood whether interactions of MT1-MMP with CD44 and ERM proteins occur during secretion or upon cell surface expression at lamellipodia. As discussed above, one of the functional activation processes of MT1-MMP is homo-dimerization, and the MT1-MMP homodimer has been shown to be highly localized at membrane ruffles [55]. Thus, MT1-MMP at the lamellipodia is functionally active for collagen degradation and proMMP-2 activation.

Lamellipodia have been observed and characterized in 2D cell culture conditions, but in the 3D migration process, cells are considered to form small lamellipodia-like membrane structures at the leading edge [84]. However, the implication of MT1-MMP localization at lamellipodia in the 3D migration process is not clearly understood.

### 3.2. Localization at Focal Adhesions

Focal adhesions (FAs) are formed by clustered integrins interacting with the ECM on the outside of cells and form a complex at the C-terminus of the beta integrin subunit with focal adhesion kinase, talin, paxillin, and vinculin. This complex connects integrins to the actin cytoskeleton within the cells [85]. The plasma membrane is closest to the ECM at FAs, and FAs transmit mechanical tension from the outside to the inside of cells [85]. During cell migration on a 2D substratum, FAs act as crucial cell adhesion structures [85]. However, FAs can be a potential precursor of the leading edge of invading cells, as it has been shown that MT1-MMP accumulates at FAs [11,59,60]. Moreover, abrogating MT1-MMP localization at FAs inhibited cellular invasion [11,71]. It was found that MT1-MMP destabilized stable FA formation by degrading ECM at FAs [11,59]. The MT-Loop region of MT1-MMP (^163^PYAYIREG^170^) is essential for its FA localization, and the deletion of the MT-Loop inhibits cell invasion and ECM degradation at the FA [11]. Since treating cells with a specific antibody against MT-Loop mimics the phenotype of the MT-Loop deletion mutant of MT1-MMP, it is most likely that the MT-Loop region is the interface of molecular interactions required for MT1-MMP localization at the focal adhesion [11]. The requirement of MT-Loop for the degradation of substratum at FAs has been shown in HT1080, A431, and COS7 cells [11]. These findings indicate that FAs can be considered a precursor of the leading edge of invading cells.

**Figure 3:**
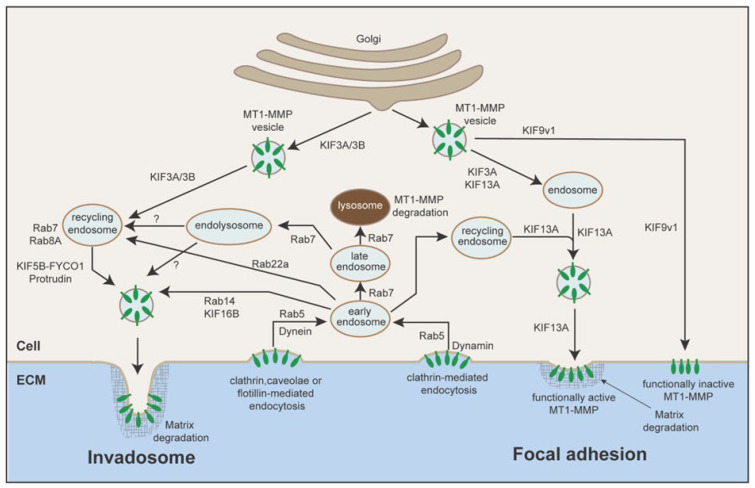
MT1-MMP trafficking to membrane structures. **Invadosome:** Upon synthesis at the ER and maturation in the Golgi, MT1-MMP is sorted into secretory vesicles. KIF3A and KIF5B were shown to be crucial KIFs for vesicle transport to the invadosome. Protrudin makes contact sites with Rab7 and PI3P–positive late endosomes, containing MT1-MMP. Protrudin hands over Rab7-binding KIF5 adaptor protein FYCO1, enabling the transport of MT1-MMP-containing vesicles along microtubules toward invadopodia. MT1-MMP function on the cell surface is maintained by replacing old MT1-MMP with newly synthesized molecules, and old cell surface MT1-MMP needs to be endocytosed. Flotillin-dependent endocytosis of MT1-MMP into early endosomes, mediated by dynein and Rab5, has been shown to be crucial for invadopodia-mediated matrix degradation. Rab7 further transports MT1-MMP-containing vesicles from the early endosome to late endosomes. Some MT1-MMP is transported to the lysosome for degradation, but some is transported to and stored in endolysosomes without degradation for efficient recycling to endosomes. It is not clear if MT1-MMP in the endolysosome is recycled back to the cell surface through the recycling endosome or directly transported from the endolysosome. It has been shown that Rab14 regulates fast recycling alongside KIF16B, while Rab22a regulates slow recycling through recycling endosomes. It is not clear if these Rab small GTPases are involved in recycling through endolysosomes. **Focal adhesion:** Upon synthesis at the ER and maturation in the Golgi, MT1-MMP is sorted to secretory vesicles. Both KIF3A and KIF13 are necessary to transport the vesicle to the endosome. From the endosome, KIF13A alone traffics the MT1-MMP-containing vesicles to the focal adhesion. KIF9v1 competes with KIF3A to transport MT1-MMP vesicles to different membrane domains, where MT1-MMP is not functionally active. Clathrin-dependent endocytosis was found to be crucial for MT1-MMP-mediated cell invasion at focal adhesions. Dynamin was shown to play a role in this endocytic pathway. Some endocytosed MT1-MMP can be transported to the endolysosome for degradation, and some is recycled back to the cell surface through the recycling endosome. Rab small GTPases involved in this model of vesicle transport may be the same as those involved in MT1-MMP transport to the invadosome, but this is currently unknown.

MT1-MMP localization to FAs is accomplished by targeted vesicle transport along microtubules by kinesin superfamily motor proteins (KIFs) [71]. Upon translation of MT1-MMP at the rough endoplasmic reticulum and maturation in the Golgi apparatus, the trans-Golgi network is the last station of the MT1-MMP secretory pathway. KIF3A and KIF13A coordinate the MT1-MMP-containing vesicle transport from the trans-Golgi to the endosome. From the endosome, KIF13A continues to transport MT1-MMP-containing vesicles to the leading edge [71]. There is an alternative vesicle transport pathway by KIF9 variant 1 (KIF9v1). KIF9v1 seems to compete for MT1-MMP vesicles with KIF3A and KIF13A and transport them to the cell surface away from the leading edge, where MT1-MMP seems to be in a functionally inactive form (Figure 3) [71]. Knockdown of KIF3A or KIF13A does not reduce the overall cell surface level of MT1-MMP, but it significantly decreases the matrix degradation and collagen invasion of HT1080 cells [71], suggesting that localizing MT1-MMP at FAs is a crucial step for cancer cells to invade. However, the detailed mechanisms of how these KIFs specifically recognize MT1-MMP-containing vesicles and how they coordinate with other cell migration machinery are not known.

### 3.3. Localization at Invadopodia and Podosomes

Invadopodia and podosomes are collectively called invadosomes, which are membrane structures found in cells capable of proteolytic migration [62]. MT1-MMP has been shown to localize to both invadopodia and podosomes, and significant amounts of investigation have been carried out to understand these localization mechanisms.

Invadopodia are membrane protrusions of around 0.5–3.0 μm in width and 2–8 μm in length, which are found in invasive cancer cells [61]. They form at the ventral side of cultured cells, perpendicular to lamellipodia. Molecularly, invadopodia are defined as membrane structures colocalizing F-actin, scaffold protein Tks5 (tyrosine kinase substrates with 5 SH3 domain), the actin-regulating molecule cortactin, neural Wiskott–Aldrich syndrome protein N-WASP, and MT1-MMP [62].

Invadopodia have been identified in multiple cancer subtypes in culture [73,78,86,87]. However, some other cell types such as human cervical cancer HeLa cells, human fibrosarcoma HT1080 cells, and human squamous carcinoma A431 cells may not use invadopodia to degrade matrix during invasion (Table 1). The cytoplasmic domain of MT1-MMP, a crucial domain of MT1-MMP for invadopodia localization [88], was shown to be dispensable for matrix degradation in these cells [11]. In 3D culture conditions, it has been shown that MDA-MB-231 cells in a high-density fibrillar collagen matrix use invadopodia to degrade collagen [89]. While invadopodia have been extensively studied in breast cancer cells such as MDA-MB-231, as these cells exclusively use invadopodia for invasion, it is still not clear what other cells exclusively use invadopodia for invasion.

Podosomes are membrane structures with a width of around 0.4 μm and a length of 1 μm, found on the ventral part of the cell membrane [61]. Originally discovered in the 1980s in Rous sarcoma-transformed fibroblasts, they are often described as a counterpart membrane structure to invadopodia, as they tend to be seen in non-cancerous migratory cells such as osteoclasts [90]. Podosomes are much smaller than invadopodia, and while cells will usually only have a few invadopodia, they can have hundreds of podosomes [61]. Due to their small height, podosomes do not protrude into the ECM [61]. Podosomes have not yet been observed in vivo [91], although some evidence of podosome-like structures have been identified in macrophages [61,92,93,94].

Podosomes have an actin core surrounded by a ring of adhesion plaque proteins and kinases. Adhesion plaque proteins include vinculin, talin, kindlin, and associated kinases include c-Src, PI3K and PKC [95]. In contrast, invadopodia do not have this ring structure surrounding the actin core. There are different arrangements of podosomes in different cell types. Osteoclasts can form a ring of podosomes around the edge of the cell or horseshoe-shaped podosome accumulations. Macrophages tend to have an even distribution of podosome clusters across the ventral surface, whereas smooth muscle cells have podosomes localized to the edges of the cell [96]. Endothelial cells form ‘rosette’ podosome clusters [96].

MT1-MMP has been shown to localize to invadosomes through targeted vesicle transport, mediated by KIFs. In macrophages, it has been shown that specific KIFs involved in MT1-MMP transport to the podosomes include KIF5B and KIF3A/B [97]. The dynein/dynactin complex mediates retrograde transport of the same vesicles away from the cell surface after endocytosis of MT1-MMP [97]. KIF3A and KIF5B have also been implicated in MT1-MMP transport to invadopodia in invasive breast cancer cells [98]. In addition, protrudin is required for MT1-MMP localization to invadosomes, as protrudin binds to PI3P-positive late endosomes containing MT1-MMP [99]. MT1-MMP localization to podosomes has mostly been studied in the context of macrophages.

It has been shown that the cytoplasmic tail of MT1-MMP is crucial for invadopodia localization [78,88]. However, this mechanism is not well understood. Recently, it has been shown that MDA-MB-231 cells shed microvesicles containing MT1-MMP, and these microvesicles can transfer MT1-MMP to MT1-MMP-negative HeLa cells, making them invasive [100]. In this microvesicle shedding process, the interaction of Tks5 with MT1-MMP cytosolic tail at the endosome was shown to be crucial [100]. This suggests that Tks5, a crucial component of invadopodia, indeed interacts with the MT1-MMP cytosolic tail, and may explain the requirement of the cytosolic tail for MT1-MMP localization at invadopodia.

The Rab small GTPases play crucial roles in intracellular vesicle transport [101], and some of the Rabs have been identified to play roles in MT1-MMP recycling and subsequent localization to endosomes. Rab4 and Rab5a were shown to be essential in MT1-MMP endocytosis and recycling. Silencing these small GTPase genes prevented fast MT1-MMP recycling in Hela cells. Knockdown of Rab5 was shown to specifically prevent MT1-MMP endocytosis [102]. In macrophages, knockdown of Rab5a increased cell surface level of MT1-MMP [93], further supporting the role of Rab5 in MT1-MMP endocytosis.

Rab14 and Rab16B have been shown to associate with MT1-MMP in the early endosome and are thought to be involved with MT1-MMP fast recycling back to the cell surface [93,103]. Knockdown of Rab14 or Rab16B reduced expression of MT1-MMP on the macrophage cell surface [103]. Rab22a was thought to be involved in the slow recycling of MT1-MMP, but siRNA knockdown of Rab22a did not affect macrophage proteolytic migration in a 3D invasion assay [93]. Rab7 was shown to localize at the late endosome, endolysosome, and lysosome during MT1-MMP internalization, and to play an important role in endosome maturation [67,104]. Knockdown of FYCO1, a KIF5B adaptor protein which binds to Rab7, has also been shown to reduce MT1-MMP surface expression [99]. Rab8A has also been implicated in MT1-MMP transport from the recycling endosome back to invadosomes at the cell surface [93]. Knockdown of Rab8A decreases the expression of MT1-MMP on the cell surface [93].

The protrudin-positive ER was shown to associate with Rab7-positive endosomes containing MT1-MMP [99]. Protrudin loads late endosomes and lysosomes with KIF5B, which may then recycle these vesicles back to the plasma membrane [99]. Thus, MT1-MMP localization is determined by KIFs, Rab small GTPase families, and other molecules. Figure 3 summarizes the transport mechanisms of MT1-MMP to the focal adhesion as well as to invadosomes.

## 4. Conclusions and Future Prospects

MT1-MMP has been considered a promising therapeutic target for cancers and other tissue-destructive diseases, and DX-2400, a highly selective MT1-MMP inhibitory antibody, was developed [105]. However, despite its promising preclinical data [105,106,107], DX-2400 could not proceed to clinical trial. This is due to the previous failure of clinical trials on broad-spectrum small-molecule MMP inhibitors caused by the side effects of musculoskeletal syndrome and no efficacy [108,109]. The failures are considered to be due to the inhibition of off-target metalloproteinases and caused a huge financial loss to pharmaceutical companies. This made an investment in another “MMP inhibitor”, DX-2400, too much of a financial risk for companies to consider despite the specific nature and promising data of this MT1-MMP inhibitor. However, since MT1-MMP-mediated cellular invasion can be inhibited by modifying its regulatory mechanisms, it may be possible to target MT1-MMP-mediated invasion without inhibiting the catalytic activity of MT1-MMP, avoiding the side effects seen in previous clinical trials.

Research over recent years has deepened our understanding of MT1-MMP transport in migratory cells. Localization of MT1-MMP to the leading edge, clearance of the enzyme from the cell surface by endocytosis, and recycling back to the cell surface are essential mechanisms in MT1-MMP-mediated cell migration and invasion. Thus, developing drugs that specifically target MT1-MMP localization at the leading edge could be effective anti-invasion/tissue destruction therapies while avoiding the toxicity of broad-spectrum MMP inhibitors [108,109]. Despite recent advances in immunotherapies in oncology and rheumatic diseases, no treatment has 100% efficacy, and there are always non-responding patients [110,111]. Therefore, there is a need to develop alternative treatments for non-responders to support current immunotherapeutic treatment, and inhibition of MT1-MMP-mediated cellular invasion could be a potentially promising means to complement current immunotherapies [88,110].

Targeted transport of MT1-MMP-containing vesicles to the plasma membrane by KIFs is a crucial step that regulates the cell-surface level and localization of MT1-MMP to the leading edge. Currently, targeting KIFs for blocking cellular invasion has not been explored as a therapeutic avenue, as most attempts to target KIFs clinically have been within the group of KIFs involved in mitosis [112]. Rab GTPases could also be a potential druggable target to prevent cellular invasion. However, due to their highly conserved structure, it is difficult to target single Rab GTPases with either small molecules or antibodies [113]. Although there has been some success in preliminary gene therapy trials, these treatments have many off-target effects that would need to be addressed before clinical use [113]. As discussed above, much of the research on the MT1-MMP trafficking mechanism has been conducted on invadosomes. However, different cell types seem to employ different mechanisms. MDA-MB-231 cells exclusively use invadopodia for invasion, whereas other cancer cell types, such as HT1080, A431, and HeLa cells, use other membrane structures such as focal adhesions. The mechanism of MT1-MMP localization to invadopodia differs from MT1-MMP localization to focal adhesions and other associated leading-edge membrane structures. Therefore, understanding the leading-edge localization mechanisms of MT1-MMP across different cancer types would be of significant value. Further investigation of the mechanisms of MT1-MMP localization may result in the identification of novel targets that could be used to inhibit MT1-MMP-mediated cellular invasion and tissue destruction in the future.

## Figures and Tables

**Table 1:** Cells that utilize different membrane structures for ECM degradation: the cell types/cell lines are listed for each membrane structure cells use for ECM degradation. Supporting references are also indicated for each cell type/cell line.

Membrane Structures	Cell Types	Cell Line	References
Focal adhesion	Fibroblasts	-	[71]
	Fibrosarcoma	HT1080	[11,58]
	Pancreatic cancer	PANC-1	[11,59]
	Squamous cell carcinoma	A431	[60]
	Cervical cancer	HeLa	[11]
Invadopodia	Squamous cell carcinoma	SCC61	[72]
	Breast cancer	MDA-MB-231	[73]
		4T1	[74]
		CA1d	[75]
	Bladder cancer	-	[76]
	Melanoma	RPMI7951	[77,78]
		A375MM	[79]
Podosomes	Macrophage	-	[61]
	Osteoclasts	-	[61]
	Endothelial cells	-	[80]
	Transformed fibroblasts (src or v-src)	-	[61]

## Data Availability

No new data were created or analyzed in this study. Data sharing is not applicable to this article.
